# Symptomatic cerebral oedema during treatment of diabetic ketoacidosis: effect of adjuvant octreotide infusion

**DOI:** 10.1186/1758-5996-2-56

**Published:** 2010-08-19

**Authors:** Ora Seewi, Anne Vierzig, Bernhard Roth, Eckhard Schönau

**Affiliations:** 1 Uniklinik Köln, Clinic for General Paediatrics, University Hospital of Cologne, Germany

## Abstract

**Introduction:**

A potentially lethal complication of diabetic ketoacidosis (DKA) in children is brain oedema, whether caused by DKA itself or by the therapeutic infusion of insulin and fluids.

**Case presentation:**

A 10-year old previously healthy boy with DKA became unconscious and apnoeic due to cerebral oedema (confirmed by abnormal EEG and CT-scan) during treatment with intravenous fluids (36 ml/h) and insulin (0.1 units/kg/h). He was intubated and artificially ventilated, without impact on EEG and CT-scan. Subsequently, adjuvant infusion of octreotide was applied (3.5 μg/kg/h), suppressing growth hormone (GH) and IGF-1 production and necessitating the insulin dose to be reduced to 0.05 - 0.025 units/kg/h. The brain oedema improved and the boy made a full recovery.

**Conclusion:**

Co-therapy with octreotide was associated with a favourable outcome in the present patient with DKA and cerebral oedema. Whether this could be ascribed to the effects of octreotide on the insulin requirement or on the GH/IGF-axis remains to be elucidated.

## Introduction

Cerebral oedema is the most feared complication of DKA. The pathogenesis appears complex and is poorly understood [1]. According to a recent working hypothesis, dehydration and hypocapnia diminish cerebral perfusion, resulting in mild brain ischaemia and subsequent cytotoxic and vasogenic cerebral oedema [1]. In this context, hypoxia-induced VEGF activity may play a role [2]. Insulin treatment might also contribute, for example via its sodium-retaining effects, or by its effects on the growth hormone (GH)/insulin-like growth factor (IGF)-axis [3]. Insulin increases serum IGF-1 and decreases IGFBP-1, thereby increasing free IGF-1 activity. IGF-1 increases capillary permeability [4] and oedema formation [5,6], probably via increasing the activity of VEGF [7,8]. As insulin enters the brain [9], these effects may - on a local level-contribute to cerebral oedema during DKA. Somatostatin, which counteracts some insulin effects on the GH/IGF-axis [10] and lowers free IGF-1 in particular [11], was successfully applied in complicated DKA by Bosnak et al. [12]. These authors added a somatostatin infusion to the standard DKA therapy in two unconscious children with DKA who subsequently regained consciousness within 3-4 hours. The therapeutic mechanism of somatostatin in this condition remains obscure. We provide additional evidence for the use of somatostatin to improve the outcome of cerebral oedema in childhood DKA.

## Case presentation

A previously healthy boy of 10 years (estimated body weight 25 kg) was admitted acutely after vomiting for 8 hours, preceded by polyuria, polydipsia, weight loss and malaise for 3 days. He was conscious, but drowsy (Glasgow Coma Scale score 12). A diagnosis of diabetic ketoacidosis was made because of hyperglycaemia (1329 mg/dl) and metabolic acidosis (pH 7.07, base excess-19.7 mmol/l). He was severely dehydrated and displayed Kussmaul breathing. Immediately upon admission he received a s.c. injection of 2.5 units of regular human insulin, followed by continuous intravenous infusion of regular insulin at a dose of 0.1 U/kg/h. Simultaneously, an intravenous bolus infusion of 250 ml of 0.6% saline was administered, followed by continuous intravenous infusion of 0.9% saline and 0.45% NaCl plus 5% glucose (36 ml/h). The baseline blood chemistry showed pre-renal kidney failure (serum creatinine 3.75 mg/dl, urea 151 mg/dl, uric acid 20.4 mg/dl), circulatory insufficiency (lactate 3.2 mmol/l) and dehydration (measured osmolality 444 mosmol/kg, haematocrit 54%). Furthermore, leukocytosis (15.77× 10-9/l) and a reduced serum chloride of 88 mmol/l were noted. The serum sodium, potassium, phosphate, calcium, magnesium, CRP, liver enzymes and clotting factors were normal. The serum GH was elevated (7.5 ng/ml), while the IGF-1 was below the detection limit (< 10 ng/ml), consistent with acquired GH insensitivity. After 10 hours of treatment with insulin and potassium-supplemented fluids, the acidosis had almost been cured (pH 7.39, base excess -2.8 mmol/l), and glycaemia was more than halved (549 mg/dl). The serum osmolality had dropped to 380 mosmol/l, the GH had fallen to 2 ng/ml, and the IGF-1 had risen to 23 ng/ml (suggestive of an immediate, insulin-induced rise in circulating IGF-1 [13,14]). See Table 1 for a summary of the laboratory data.

**Table 1:** Laboratory data before, during and after treatment of DKA

Blood chemistry	Time (h) after admission			
	0	10	24	44
Glucose (mg/dl)	1392	549	214	114
PH	7.07	7.39	7.33	7.35
HCO_3 _(mmol/l)	9.2	22.5	20.3	21.7
Base excess (mmol/l)	-19.7	-2.8	-4.9	-3.1
Na+ (mmol/l)	143	162	154	158
K+ (mmol/l)	5.8	4.9	5.6	3.5
Osmolality (mosmol/kg)	444	380	n.a*	342
Creatinine (mg/dl)	3.75	1.86	1.66	1.24
Haematocrit (%)	54	45	44	40
Lactate (mmol/l)	3.2	1.5	1.3	1.1
GH (ng/ml)	7.5	n.a*	2.0	< 0.16
IGF-1 (ng/ml)	< 10	n.a*	23	< 10
*not analysed				

Reference values for a 10 year old male child

Fasting blood glucose: 60-110 mg/dl;

arterial pH 7.4;

arterial HCO3 31 mmol/l;

arterial base excess +5 mmol/l;

arterial serum lactate 1.0-1.6 mmol/l;

venous serum natrium 130-145 mmol/l;

venous serum potassium 3.2-5.4 mmol/l;

venous serum osmolality 280-296 mosmol/kg;

venous serum creatinine < 0.9 mg/dl;

venous blood haematocrit 34-46%;

venous serum random GH < 4 ng/ml;

venous serum IGF-1: 159-300 ng/ml.

**Figure 1:**
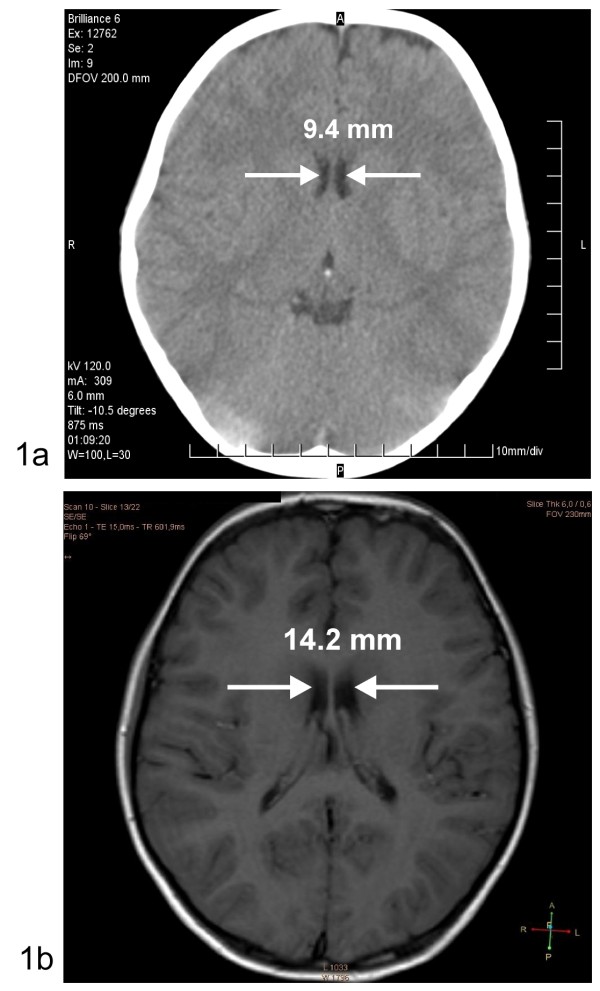
**Intercaudate diameter: (a) according to Glaser et al**. [15], during DKA. Computed tomography without contrast media; (b) after full recovery from DKA. Magnetic resonance imaging (T1 weighted) without contrast media.

However, the boy became agitated and febrile and exhibited apnoeic phases indicative of incipient cerebral oedema. He was intubated and artificially ventilated under sedation with propofol and later midazolam, ketamin and fentanyl. Repeated CT-scans of the brain were unhelpful (in retrospect, the intercaudate diameter was narrowed by 4.8 mm (see Fig 1a)). Electroencephalography (EEG), however, showed a slow basic rhythm consistent with the manifestation of encephalopathy. At this point, DKA treatment failure was considered and it was, therefore, decided to try infusion of the somatostatin-analogue octreotide (3.5 μg/kg/h), according to Bosnak et al. [12]. In response to the octreotide infusion, the insulin requirement decreased while serum GH and IGF-1 decreased below the detection limits, and serum osmolality decreased to 342 mosmol/l. After 20 hours of co-therapy with octreotide, extubation was successfully attempted. The octreotide infusion was tapered off and the insulin infusion was changed to subcutaneous injections. The patient made a full recovery without neurological deficits. The treatment is summarized in Table 2.

**Table 2:** Treatment items before, during and after treatment of DKA

Treatment items	Time (h) after admission			
	0	10	24	44
net fluid gain* (ml/kg)	10	13	83	116
GCS**score (3-15)	12	8	not applicable	15
Artificial ventilation -/+	---	-- < ++++	++++++++++	++++ > ---
Octreotide infusion (μg/kg/h)	0	0	3.5	1.8
Insulin infusion (U/kg/h)	0.1	0.1	0.05	0.025

*total intravenous fluids minus urinary output; **GCS = Glasgow Coma Scale

The transient increases in liver enzymes had normalized at the time of discharge, as had all of the other laboratory abnormalities, except for an HbA1c of 12% (normal 4-6%). The fasting serum GH was 0.16 ng/ml and the IGF-1 was 132 ng/ml, suggesting normalized GH sensitivity. The patient was discharged with 23 U of NPH, and 20 U of regular human insulin per day. One week after discharge, the intercaudate diameter was no longer narrowed on cranial MRI (Fig. 1b).

## Discussion

The case of our patient is comparable to those reported by Bosnak et al. [12], in that a comatose child with DKA receiving co-therapy with the somatostatin analogue octreotide regained consciousness. However, the patients of Bosnak et al. were already unconscious before the onset of treatment, and it was claimed that they did not have brain oedema [12]. By contrast, our patient lost consciousness during insulin and fluid substitution and subsequently developed cerebral oedema (according to the established criteria [15], see Fig. 1a, b).

Fluid overload, the use of sodium bicarbonate or prolonged acidosis are unlikely to have caused cerebral oedema in our patient as sodium bicarbonate administration had been withheld and the net fluid gain was only 326 ml (13 ml/kg) during the first 10 hours, and the acidosis was almost cured when cerebral oedema occurred. However, hyperosmolality had dropped considerably (from 444 to 380 mosmol/l) at that time point, probably because 0.6% rather than 0.9% saline was given without consideration of the current guidelines [15,16]. This might have contributed to the development of brain oedema, although consciousness was regained later despite a further drop in osmolality (from 380 to 342 mosmol/l). Thus, additional factors deserve consideration, for example insulin [17] and its effects on IGF-1.

In insulin-naive untreated type-1 diabetic children with DKA, serum insulin concentrations are < 5 μU/ml (unpublished observation) and increase upon therapeutic intravenous insulin infusion at a rate of 0.1 U/kg/h to approximately 60 - 100 μU/ml [13,18-20]. Such serum insulin levels could also have been assumed in our patient at the time of developing brain oedema (unfortunately the insulin levels were not measured). Continuous hyperinsulinaemia such as this overcomes insulin resistance, shuts off glycolysis, gluconeogenesis and ketogenesis, half-maximally increases glucose uptake[20], and furthermore substantially increases circulating IGF-1 within a few hours [13,14]. A rapid increase in circulating IGF-1 from below the normal range to the upper normal range [13,14] may contribute to the formation of oedema in general, and to cerebral oedema during DKA treatment, which is consistent with previous reports [4-6].

During co-therapy with octreotide, the insulin infusion rate had to be reduced from 0.1 U/kg/h to 0.05 U/kg/h (as with the patients of Bosnak et al.), probably due to the inhibition of glucagon and GH secretions. An insulin infusion rate this low is associated with a serum insulin concentration of 10-15 μU/ml, and avoidance of the immediate upregulation of serum IGF-1 during DKA treatment (unpublished observation). Contrary to current guidelines [16,21] it was recently shown that low rate insulin infusion may be particularly safe in DKA [17,22].

In our patient, octreotide completely inhibited the insulin-induced surge of circulating IGF-1, most likely through the inhibition of GH secretion. Moreover, octreotide must have affected the serum IGFBP-1 and free IGF-1 in our patient (not measured). In severe insulin deficiency, for example in diabetic ketoacidosis, IGFBP-1 is upregulated while hepatic IGF-1 secretion is reduced, despite increased GH [2,13,14,23]. Insulin substitution reverses these abnormalities [2]. By contrast, octreotide administration increases circulating IGFBP-1 [10,11] and thereby reduces free IGF-1 [11]; moreover, it antagonizes the stimulating effect of GH on IGF-1 generation in various tissues, e.g. the brain. Both processes may have contributed to the resolution of cerebral oedema in our patient, and to the regaining of consciousness in the patients of Bosnak et al., as resolution of diabetic macula oedema was observed in response to octreotide administration [24,25].

In summary, we report for the first time a successful adjunctive therapy with octreotide in a patient with DKA and proven cerebral oedema. Although we cannot prove a cause-effect relationship, we believe that the potential of octreotide in this condition warrants further study.

## Abbreviations

CT: computed tomography; DKA: diabetic ketoacidosis; GH: growth hormone; IGF-1: insulin-like growth factor 1; IGFBP-1: insulin-like growth factor binding protein 1; MRI: magnetic resonance imaging; VEGF: vascular endothelial growth factor

## Consent

Written consent was obtained from the patient's parents for publication of this case report and any accompanying images. A copy of the written consent is available for review by the Editor-in-Chief of this journal.

## Competing interests

The authors declare that they have no competing interests.

## Authors' contributions

OS analyzed and interpreted the patient data and was major contributor in writing the manuscript. AV and BR were responsible for the intensive care of the patient. ES was involved in drafting the paper. All authors read and approved the final manuscript.
